# Integrating the 5-SENSE Score for Patient Selection in Vagus Nerve Stimulation for Drug-Resistant Epilepsy

**DOI:** 10.7759/cureus.68003

**Published:** 2024-08-28

**Authors:** Flavius Iuliu Urian, Radu Eugen Rizea, Horia Petre Costin, Antonio-Daniel Corlatescu, Gabriel Iacob, Alexandru Vlad Ciurea

**Affiliations:** 1 Department of Neurosurgery, University Emergency Hospital, Bucharest, ROU; 2 Department of Neurosurgery, Clinical Emergency Hospital “Bagdasar-Arseni”, Bucharest, ROU; 3 Department of Neurosurgery, Carol Davila University of Medicine and Pharmacy, Bucharest, ROU

**Keywords:** vagal nerve stimulation, seizure frequency reduction, stereo-electro-encephalography, electrophysiology, neuroimaging, focal epilepsy, 5-sense score

## Abstract

Addressing the challenge of drug-resistant epilepsy, our study offers a novel perspective by retrospectively applying the 5-SENSE score, initially created for stereoelectroencephalography (SEEG) planning, to evaluate its predictive value in patients undergoing vagus nerve stimulation (VNS) therapy.

We conducted a comprehensive preoperative diagnostic work-up, including computed tomography (CT), magnetic resonance imaging (MRI), positron emission tomography-CT (PET-CT), video-electroencephalogram (video-EEG), and clinical semiology. We then stratified 76 patients into three groups - low, moderate, and high focality - based on the focality of the seizure-onset zone. Such stratification was made to check the scoring ability in predicting VNS therapy seizure reduction.

Our findings demonstrate an association between the extent of focality at the seizure-onset zone and the effectiveness of VNS, which may help to define the role of the 5-SENSE score in patient selection for VNS. This high dispersion of responses in the group with high focality reinforces the idea that outcome estimation is difficult and argues for an individualized strategy in the treatment of drug-resistant epilepsy.

A study at the level of the 5-SENSE score indicates the importance of detailed preoperative assessments that may better optimize selection for VNS therapy and further improve clinical outcomes.

## Introduction

A major advance in the management of epilepsy is the development of the 5-SENSE score, which improves patient stratification for stereoelectroencephalography (SEEG) and helps identify potential candidates for vagus nerve stimulation (VNS). This nuanced approach to seizure onset zone focality addresses the gap in cases where non-invasive evaluation lacks definitive clarity [1-3].

The 5-SENSE score includes five predictive variables: focal lesion on structural Magnetic resonance imaging (MRI), absence of bilateral independent spikes on scalp electroencephalogram (EEG), localizing neuropsychological deficit, strongly localizing semiology, and regional ictal scalp EEG onset [4].

Multicenter studies validated the 5-SENSE, confirming its specificity, sensitivity, and clinical relevance. It effectively identifies patients unsuitable for stereoelectroencephalography (SEEG) due to nonfocal or undetectable seizure onset zones. Recent research highlights pre-ictal seizure detection via electroencephalogram (EEG) and the role of machine learning in improving seizure prediction and VNS therapy [5]. Better predictive accuracy is achieved by assessing seizure type, etiology, EEG characteristics, and heart rate variability (HRV). Advances in noninvasive VNS systems that detect seizures underscore the importance of accurate prediction and patient selection [6-8]. Visual evoked potential (VEP) modeling and SEEG have enhanced understanding of epileptic networks, improving diagnostics and therapy for better outcomes [9-12].

## Materials and methods

Study objective

The retrospective study primarily aims to assess the effectiveness of VNS in managing refractory epilepsy by evaluating the correlation between the preoperative diagnostics-based 5-SENSE score and seizure frequency reduction. Additionally, it seeks to identify which focality group (low, moderate, and high) experiences the greatest reduction in seizure frequency following VNS therapy.

Study design

A retrospective, descriptive, and exploratory analysis was conducted on 76 patients with drug-resistant epilepsy treated at the Epileptology Unit of the Neurology Department and Neurosurgical Department II at University Emergency Hospital Bucharest, Romania, between 2022 and 2024. Patients with a predefined multifaceted preoperative evaluation that included the 5-SENSE score were selected after imaging in the form of computed tomography (CT), magnetic resonance imaging (MRI), interictal positron emission tomography-CT (PET-CT) scans, video-electroencephalogram (video-EEG), semiology of seizures, and classification of epilepsy according to the International League Against Epilepsy (ILAE)-2017 guidelines. In this respect, the outcomes of patients after VNS therapy were analyzed based on data collected at the last follow-up visit.

Five-SENSE score evaluation

5-SENSE score is one of the core parts of the study and is the basis for the classification of patients into three focality groups low (0-1 points), moderate (2-3 points), and high focality (4-5 points) according to the localization of seizure-onset zone. It was developed after a detailed analysis of the following parameters: focal lesions on structural MRI, absence of bilateral independent spikes on the scalp EEG, localizing neuropsychological deficits, strongly localizing semiology, and regional ictal scalp EEG onset.

Outcome parameters for VNS efficacy

The efficacy of VNS therapy has been measured very cautiously, taking into consideration the percentage reduction in monthly seizure frequency, calculated as the difference between post-VNS and pre-VNS median monthly seizure frequency. The approach allowed estimation of the treatment influence in the form of a completely clear percentage change and, therefore, represented the most exact quantitative measure of VNS outcomes. This was an easy procedure that made standard comparison possible among patients whose frequencies of baseline seizures varied. This percentage change orientation allowed us to summarize the complex influence of VNS in one readily understandable metric that gives a clear perception of the direct impact of therapy on seizure reduction.

Statistics

Our key statistical model was the Kruskal-Wallis test, a nonparametric test by design, given that the changes in the frequency of seizures were not normally distributed. The test investigated median differences in the percentage seizure reduction among the three 5-SENSE score groups. In cases where significant differences were observed, post hoc analyses were performed to identify the specific group comparisons that accounted for these differences. We also used paired t-tests to assess the mean monthly seizure reduction within the focality groups, next to the Kruskal-Wallis test. Indeed, a parametric test was applied to the data, as it provided a method for comparing pre- and post-stimulation seizure frequencies for individual patients. This approach adjusted for within-subject variability and increased the precision of our statistical inferences.

Ethical considerations

The study had been granted an ethical clearance certificate as per the guidelines of the University Emergency Hospital Bucharest. Written consent was obtained from all the participants. The study maintained very strict levels of confidentiality and ethical measures to respect the patient's rights and protect the data used in the process of carrying out the research.

## Results

Most of the study cohort falls in the 5-SENSE score low focality group (0-1 points), indicating a prevailing focality of seizure onset zones among patients implanted with VNS (Table 1).

**Table 1:** Patient categorization based on 5-SENSE scores for VNS therapy. VNS, vagus nerve stimulation

5-SENSE score groups	Interpretation	Count	Percentage
0-1 points	Low focality	45	59.21
2-3 points	Moderate focality	19	25.00
4-5 points	High focality	12	15.78

**Figure 1:**
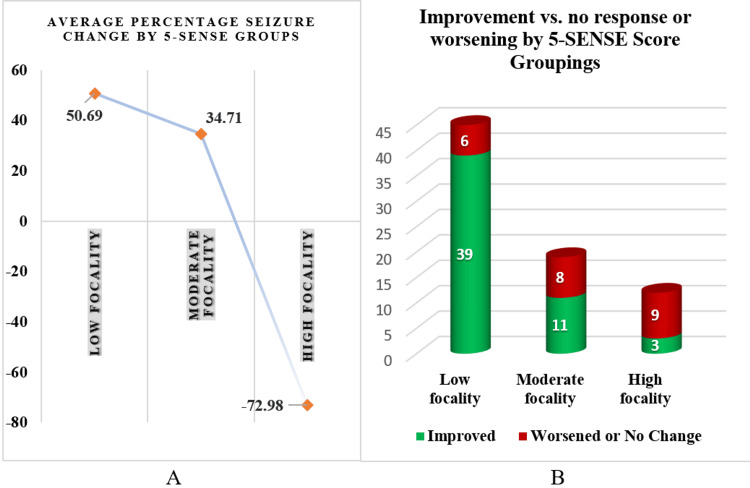
VNS therapy outcomes by 5-SENSE score categories. (A) Effects of the therapy among the 5-SENSE focality scores. (B) Patients are categorized according to improvement or worsening of their condition after VNS, showcasing the variability in patient outcomes within each focality group. VNS, vagus nerve stimulation

The results of VNS therapy varied significantly across the 5-SENSE score groups in terms of average seizure reduction (Figure 1A). Among the 45 patients in the low focality group, 39 (86.67%) experienced improvements, while 6 (13.33%) either worsened or did not improve, including one patient who showed a significant increase in seizures. These numbers provide a more quantitative view of the improvements and challenges for each focality group, revealing different patterns of response to VNS therapy (Figure 1B).

Considering the average number of seizures, the low focality group, scored at 0-1 points, showed a reduction from 41.085 seizures per month before treatment to 22.759 after, which is an average decrease of 18.33 (*P *= 0.012) seizures per month. When the percentage change in seizure frequency after VNS is considered, a different pattern emerges. The low focality group had an average seizure frequency reduction of 50.69%; however, the variability in responses ranged from an increase of 87.50% to a complete reduction, with a standard deviation of 37.75% (Table 2).

**Table 2:** Statistical evaluation of monthly seizure frequency changes by 5-SENSE focality group post-VNS. *N*, number; VNS, vagus nerve stimulation

5-sense SCORE groupings	Average monthly seizures before stimulation	Average monthly seizures after stimulation	Average decrease	Paired *t*-test results
Low focality (*N* = 45)	41.08519	22.75963	18.33	*P *= 0.0012
Moderate focality (*N* = 19)	32.88158	28.89035	3.99	*P *= 0.0388
High focality (*N* = 12)	23.5375	47.27361	-23.74	*P *= 0.3033

In terms of percentage change in seizure frequency after VNS, a different pattern emerges. For the low-focality group, the mean seizure frequency decreased by 50.69%, with individual responses ranging from an increase of 87.50% to a complete reduction; the standard deviation was 37.75%. In the moderate-focality group, the average seizure frequency decreased by 34.72%, with individual changes ranging from zero to an 83.33% reduction; the standard deviation for this group was 33.09%. The high-focality group experienced an unexpected average increase in seizure frequency by 72.98%, with a wide variability indicated by a standard deviation of 206.10%; this group included cases with a dramatic increase in seizure frequency by as much as 660.42%.

It's crucial to note that while the majority in the low focality group saw a reduction in seizure frequency, the variability in the high focality group was significant, revealing that a higher score does not uniformly predict better or worse outcomes and must be interpreted with caution.

The Kruskal-Wallis test indicated significant differences in the percentage change in seizure frequency across the 5-SENSE score groups (*P* = 0.00012). To further explore these differences, post hoc Mann-Whitney U tests were conducted.

Between the low and moderate focality groups, the difference was not statistically significant (*P* = 0.05597). A significant difference was found between the low and high focality groups (*P* = 0.000054). There was also a significant difference between the moderate and high focality groups (*P* = 0.0113).

**Figure 2:**
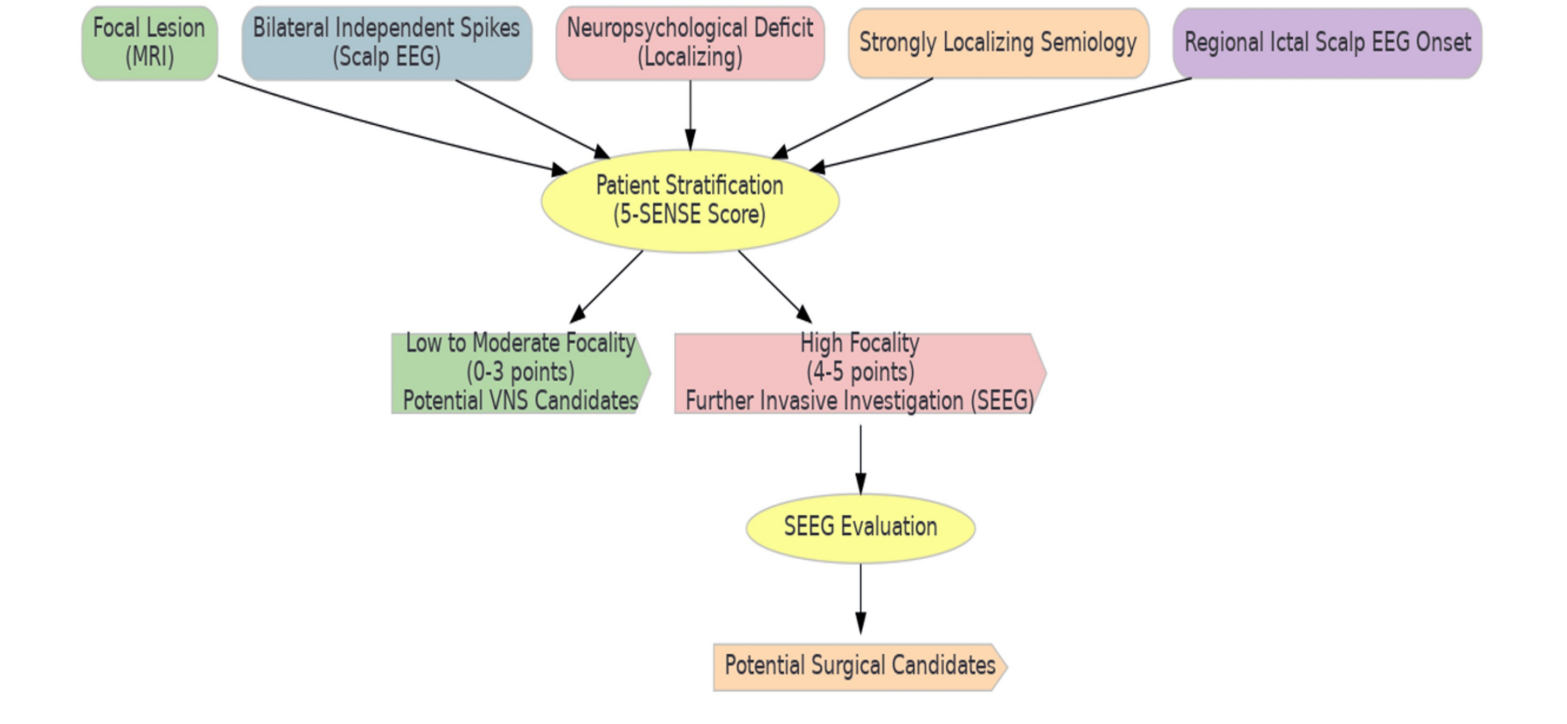
Stratification of treatment pathways in drug-resistant epilepsy using the 5-SENSE score. Image credit: All authors. SEEG, stereoelectroencephalography; VNS, vagus nerve stimulation; EEG, electroencephalogram

The 5-SENSE score, through its comprehensive evaluation of MRI findings, EEG patterns, neuropsychological assessments, and clinical semiology, offers a hypothetical framework for stratifying patients with drug-resistant epilepsy. This model suggests the potential to identify candidates who might benefit from VNS or necessitate further investigation via SEEG for surgical considerations. Emphasizing the importance of personalized medicine in epilepsy care, this approach advocates for a more nuanced patient assessment to enhance treatment decisions, presenting a conceptual pathway toward optimizing clinical outcomes (Figure 2).

## Discussion

Deciding to implant electrodes for SEEG presents a significant challenge, as the primary objective is to identify a focal seizure-onset zone, which is essential for considering potential surgical interventions. However, the success rate in accurately localizing this zone remains limited. To assist clinicians in making more informed decisions and to reduce unnecessary invasive procedures, the 5-SENSE score was developed. This score utilizes standard presurgical epilepsy evaluation data, drawing on readily available diagnostic information to predict the likelihood of SEEG successfully identifying a focal seizure-onset zone. The 5-SENSE score integrates key predictors, such as focal ictal scalp EEG onset and focal MRI lesions, which are strong indicators of a focal seizure-onset zone. Although MRI lesions alone may not always correspond to the seizure-onset zone, SEEG frequently provides additional insights that can significantly impact surgical decision-making. The score has demonstrated high specificity in external validation, making it a valuable tool for clinicians, particularly those with less experience, to estimate the probability of SEEG successfully identifying a focal seizure-onset zone and guide their treatment plans accordingly [1].

High-level VNS stimulation seems to reduce seizure frequency even in focal lesions more effectively than low-level stimulation, with a risk ratio (RR) for a 50% or greater reduction in seizure frequency of 1.73 (95% confidence interval [CI] 1.13-2.64; moderate-certainty evidence) [13].

VNS

Outcome in Lesional vs. Nonlesional Epilepsies

Generalized seizures showed the most significant response to VNS. Among patients with a history of status epilepticus (SE) before VNS implantation, 67% showed no recurrence of SE after VNS treatment [14].

Post-Epilepsy Surgery Outcomes

Surgical success rates varied by histopathology; low-grade epilepsy-associated tumors, vascular malformations, and hippocampal sclerosis had the best seizure outcomes at two years post-surgery, with 77.5%, 74.0%, and 71.5% of patients free from disabling seizures, respectively [15].

Language and Epilepsy Surgery Insights

The advanced, current surgical treatment modalities for epilepsy, especially with stereotactic laser ablation and radiofrequency ablation, have an important role in the effective treatment of epilepsy, as well as in the understanding of language networks, because of precise focal lesions in these modern surgical techniques [16]. The higher mean reduction of the seizure in the follow-up intervals was shown by a study conducted in 2021 by Zhu et al. in those undergoing deep brain stimulation of the anterior nucleus of the thalamus (ANT DBS) in comparison to the VNS group, thus opening the gateway of more patient-specific clinical features to customize the treatment modules [17]. Advanced imaging techniques and personalized modeling made it therefore possible to map epileptogenic networks, helping in surgical planning, and probably enhancing outcomes in drug-resistant epilepsy [18].

According to a study conducted by Frauscher et al., bilateral independent interictal EEG changes significantly increase the likelihood of identifying a nonfocal seizure-onset zone. Specifically, the odds ratio (OR) for a nonfocal seizure onset was 6.08 (95% CI 0.88-58.97) compared with no interictal epileptiform discharges (IEDs) and 3.26 (95% CI, 0.7-23.26) when compared with all other cases, although these results were not statistically significant (*P* = 0.08 and *P* = 0.16, respectively). These findings suggest that the presence of bilateral independent IEDs is a strong indicator of a more diffuse, rather than localized, seizure onset. Moreover, the study highlighted the relationship between neuropsychological deficits and seizure localization. A nonlocalizing neuropsychological deficit was associated with an increased probability of a nonfocal seizure onset zone (OR 0.77, 95% CI 0.26-2.05; *P* = 0.60), whereas patients with no neuropsychological deficits tended to have more focal seizure activity (OR 3.17, 95% CI 0.54-19.56; *P* = 0.20). These findings underscore the complexity of using neuropsychological assessments to predict seizure focality, with nonlocalizing deficits pointing toward a more widespread epileptogenic process, while the absence of such deficits may suggest a more focal origin [19].

The results of our study reveal that the majority of patients implanted with VNS devices fall within the low focality group, as determined by the 5-SENSE score. This categorization is based on a scoring system that evaluates MRI findings, EEG patterns, neuropsychological assessments, and clinical semiology, aiming to stratify patients with drug-resistant epilepsy into different focality groups. Specifically, 59.21% of the cohort, or 45 patients, were classified with low focality (0-1 points), demonstrating a prominent prevalence of less complex seizure onset zones among this population. The implications of these findings are significant as they suggest that patients with lower focality scores tend to exhibit better responses to VNS therapy, with 86.67% of this group showing improvement in seizure frequency. This contrasts with the high focality group (4-5 points), where only a minority of patients experienced seizure reduction, and some even saw an increase in seizure frequency. Such variability underscores the necessity of tailored treatment approaches in managing drug-resistant epilepsy and highlights the potential of the 5-SENSE score in guiding clinical decisions.

Further analysis of the seizure frequency data pre- and post-VNS implantation provides additional insights into the differential efficacy of VNS therapy across the 5-SENSE score groups. The low focality group experienced a substantial average decrease in monthly seizures, from 41.085 to 22.759, which corresponds to a 50.69% reduction in seizure frequency. Despite this overall positive outcome, there was notable variability within the group, with individual changes ranging from an 87.50% increase to a complete cessation of seizures. The moderate focality group also saw a reduction in seizures, though less pronounced, with an average decrease of 34.72% and similar variability. In stark contrast, the high focality group exhibited an unexpected average increase in seizure frequency by 72.98%, indicating that higher 5-SENSE scores might correlate with poorer responses to VNS therapy. This observation is further supported by statistical analyses, including the Kruskal-Wallis test and post hoc Mann-Whitney U tests, which confirmed significant differences between the low and high focality groups, and between the moderate and high focality groups. These findings advocate for a more individualized approach in epilepsy treatment, leveraging the 5-SENSE score to optimize patient outcomes by identifying those who are most likely to benefit from VNS therapy or who may require alternative interventions such as SEEG for surgical consideration.

While our study provides valuable insights into the application of the 5-SENSE score for patient stratification in epilepsy treatment, it is essential to recognize certain limitations that may influence the interpretation of our findings. The relatively small cohort size limits the generalizability of our results, as it may not fully represent the diverse patient populations encountered in broader clinical practice. Moreover, the retrospective nature of our analysis introduces potential biases, including selection bias and the inherent limitations of relying on existing data, which may impact the accuracy and comprehensiveness of our conclusions. These considerations highlight the need for cautious application of our findings and emphasize the importance of further research, particularly through larger, prospective studies, to validate and refine the 5-SENSE score's utility in various clinical settings.

## Conclusions

The development and validation of the 5-SENSE score represent a significant advancement in epilepsy treatment by enabling precise patient stratification for VNS therapy. This score helps identify patients for whom VNS may be less effective due to highly focal seizure onset, guiding them toward more appropriate therapeutic strategies. Our study not only validated the 5-SENSE score beyond its original SEEG context but also emphasized the potential of diagnostic tools to enhance decision-making in epilepsy treatment.

However, the variability in treatment response within the high focality group remains unexplained, suggesting that the 5-SENSE score should be applied cautiously for prognostication. These findings highlight the need for individualized treatment approaches in epilepsy. Future research should focus on understanding the factors contributing to this heterogeneity to refine the predictive value of the 5-SENSE score and tailor treatment more precisely. Such efforts will optimize standard care and improve the quality of life for patients with drug-resistant epilepsy.
